# Hollow Au–Ag Alloy Nanorices and Their Optical Properties

**DOI:** 10.3390/nano7090255

**Published:** 2017-09-04

**Authors:** Keke Yu, Xiaonan Sun, Liang Pan, Ting Liu, Anping Liu, Guo Chen, Yingzhou Huang

**Affiliations:** 1 Applied of Physics, College of Physics, Chongqing University, Chongqing 400044, China; yukeke@cqu.edu.cn (K.Y.); panliang@cqu.edu.cn (L.P.); tingliu@cqu.edu.cn (T.L.); liuanping@cqu.edu.cn (A.L.); 2 Soft Matter and Interdisciplinary Research Center, College of Physics, Chongqing University, Chongqing 400044, China; wezer@cqu.edu.cn

**Keywords:** hollow nanorice, Au–Ag alloy, surface plasmon, surface enhanced Raman spectroscopy

## Abstract

Hollow noble metal nanoparticles have excellent performance not only in surface catalysis but also in optics. In this work, the hollow Au–Ag alloy nanorices are fabricated by the galvanic replacement reaction. The dark-field spectrum points out that there is a big difference in the optical properties between the pure Ag nanorices and the hollow alloy nanorices that exhibit highly tunable localized surface plasmon resonances (LSPR) and that possess larger radiative damping, which is also indicated by the finite element method. Furthermore, the surface enhanced Raman scattering (SERS) and oxidation test indicate that hollow Au–Ag alloy nanorices show good anti-oxidation and have broad application prospects in surface-plasmon-related fields.

## 1. Introduction

Because of abundant free electrons, metal nanoparticles perform excellently in terms of optical, electric, and thermal properties, which have tremendous applications in many research fields. In recent years, the optical properties of noble metal nanoparticles have gained much interest from researchers since the light-excited collective oscillation of free electrons, i.e. surface plasmon, can confine light to a great extent on metal surfaces [1,2,3,4]. The confinement reaches a maximum when the resonance of collective oscillation occurs, generating an enormous enhanced electromagnetic field at the metal surface, and this can be applied in various fields, e.g., surface enhanced Raman scattering (SERS) [5,6,7], plasmon driven surface catalysis (PDSC) [8,9], plasmon enhanced optical activity [10], plasmonic waveguide [11,12], plasmon enhanced water splitting [13,14], plasmonic thermal effect [15,16], etc. Similar to the resonance of tuning forks, the collective oscillation of free electrons also is named localized surface plasmon resonance (LSPR), which is highly influenced by shape, size, material, and the configuration of nanoparticles [17]. Therefore, a wide variety of noble metal nanostructures have been synthesized and researched, such as nanospheres [18], nanocubes [19], nanowires [20], nanorices [21], nanoplates [22], and so on. Among them, the hollow nanostructure has been widely studied for its fascinating properties [23,24,25,26], such as its high specific surface area, high loading capacity, and low density. In previous extensive research works, the galvanic replacement reaction is adopted as a simple and convenient way to prepare hollow nanostructures, especially noble metals. Pioneered by the Xia group [27], various Au–Ag alloy nanostructures such as nanocages [28], nanotubes [29], and nanoframes [30] have been successfully prepared via galvanic replacement. During the reaction, Ag nanocrystals acting as the reducing agent are oxidized to AgCl, which have a high solubility in aqueous solution at high temperature. Meanwhile, HAuCl_4_ as the oxidizing agent are reduced to Au atoms, which are epitaxially deposited on the Ag nanocrystals. The reason is that the restriction of self-nucleation greatly promotes the deposition of Ag nanoparticles on the surface of Ag nanocrystals when the HAuCl_4_ is added to the reaction solution at a low speed [31]. It is noteworthy that the deeper reason is that lattice constant (4.078 Å and 4.086 Å for Au and Ag, respectively) and crystal structure (face-centered cubicor (fcc)) have essentially matched between Au and Ag [32]. Ultimately, the Au–Ag alloy nanostructures are obtained. In this work, via galvanic replacement reaction, Ag nanorices are used as a sacrificial template to prepare the hollow Au–Ag alloy nanorices, which not only regulate and control the optical properties of LSPR but are excellent in both chemical stability (from the segment Au) and SERS activity. To the best of our knowledge, it is the first time that hollow Au–Ag alloy nanorices have been prepared.

## 2. Result and Discussion

### 2.1. Characterization of Nanorices

**Figure 1:**
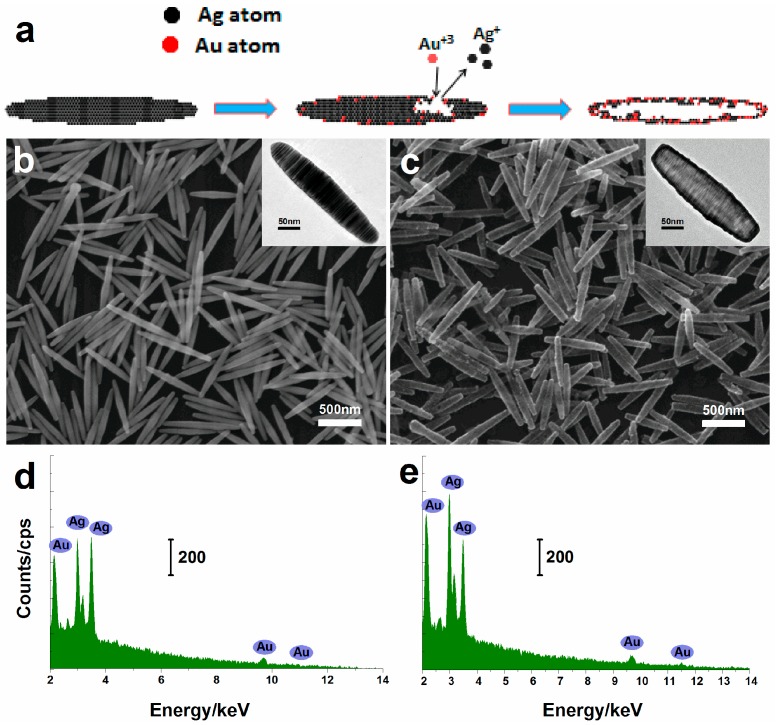
(**a**) Schematic illustration of the reaction process; (**b**,**c**) SEM and TEM (inset) of the Ag and hollow nanorices; (**d**,**e**) EDS spectra of hollow Au–Ag alloy nanorices.

Figure 1a shows the schematic illustration about the major steps for the galvanic replacement reaction. When a small amount of aqueous HAuCl_4_ was added to an Ag nanorice aqueous solution, the reaction started immediately at the situs, which possessed the highest surface energy [32]. As a result, Ag atoms were dissolved, and the Au atoms were reduced and deposited on the surface of the nanorice, which generated small holes on the surface. With the addition of HAuCl_4_, the small holes would be the primary situs for the continuous reaction. In the end, the final product was a hollow Au–Ag alloy nanorice. Figure 1b is a scanning electron microscope (SEM) image of the pristine Ag nanorices, which shows that nanorices with a uniform size distribution are obtained. The obtained product by replacement reaction is shown in Figure 1c, which indicates that the shape of the nanorices does not change. In addition, the center portion of the nanorice is lighter than its edge in the SEM image, which illustrates that the hollow structure is formed. The phenomenon is more clearly seen in the transmission electron microscope (TEM) image inset in Figure 1c. As a result, the hollow nanorices are obtained as the final product via galvanic replacement.

The hollow nanorices were investigated the energy dispersive X-ray (EDS) to check chemical composition. Figure 1d,e are collected at the border and the center of the same hollow nanorice, indicating that Ag segment is not completely replaced by Au. In other words, the shell of the hollow nanorice should be Au–Ag alloy.

### 2.2. Optical Properties of Nanorices

**Figure 2:**
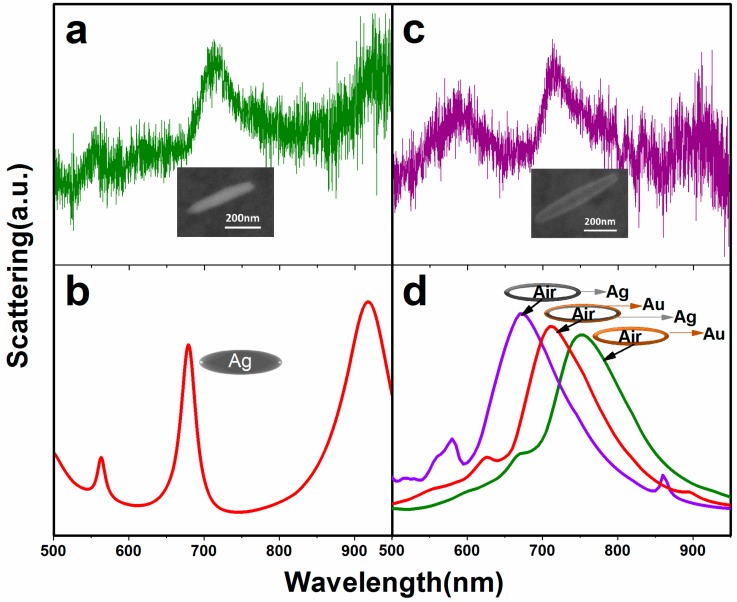
(**a**) Dark-field scattering spectra and corresponding SEM image (inset) of an Ag nanorice; (**b**) The corresponding simulation scattering spectra for the Ag nanorice with a 404 nm length and a 72 nm width; (**c**) Dark-field scattering spectra and corresponding SEM image (inset) of an Au–Ag nanorice; (**d**) The corresponding simulation scattering spectra of hollow nanorice with three different Au components.

To better understand the optical properties of this new structure (hollow Au–Ag alloy nanorice), the dark-field scattering spectra of the individual nanorice is measured. The obtained scattering spectra of individual nanorice and its corresponding SEM image are shown in Figure 2. For the Ag nanorice (with a 330 nm length and a 66 nm width), the longitudinal dipolar resonance (*n* = 1) at λ = 830 nm is apparent, and the second order resonance (*n* = 2) peak appears at 595 nm (Figure S1a). For the longer Ag nanorice (with a 404 nm length and a 75 nm width), the peaks red-shift from 830 nm and 595 nm to 920 nm and 710 nm, respectively. The third resonance peak appears in the spectra at 562 nm (Figure 2a). This phenomenon is consistent with a previous report that all multipolar plasmon peaks of Ag nanorices are red-shifted, and new peaks of higher orders appear as the increase in length for the nanorices [33]. The corresponding theoretical simulation result is shown in Figure 2b, which is found to give the best agreement with the experimental data for all multipolar plasmon modes.

The scattering spectra of a hollow Au–Ag alloy nanorice (with a 570 nm length and a 90 nm width) exhibits three higher-order plasmon resonances at approximately 583 nm, 716 nm, and 916 nm, respectively, as shown in Figure 2c. The simulated scattering spectrum and the corresponding hollow nanorice model are shown in Figure 2d. The results show that the main peak of the hollow Au–Ag alloy nanorice (the red line) is satisfactory, in agreement with the experimental result (Figure 2c). In addition, with the increase of the Au component, the plasmon resonance red-shifts and the resonant intensity decreases, which is in agreement with the simulation results of previous studies [34]. This indicates that the multipolar plasmon resonances modes of the hollow alloy nanorices can be excited and regulated by changing their Au component. In addition, the simulated scattering spectroscopy of a solid Ag nanorice under the same simulated conditions with the hollow Au–Ag alloy nanorice is shown in Figure S1b. The hollow alloy nanorice, compared with the solid Ag nanorice in the visible region, exhibits a broader peak. In other words, the hollow alloy nanorices possess larger radiative damping [33], which are more suitable acting as an effective antenna.

**Figure 3:**
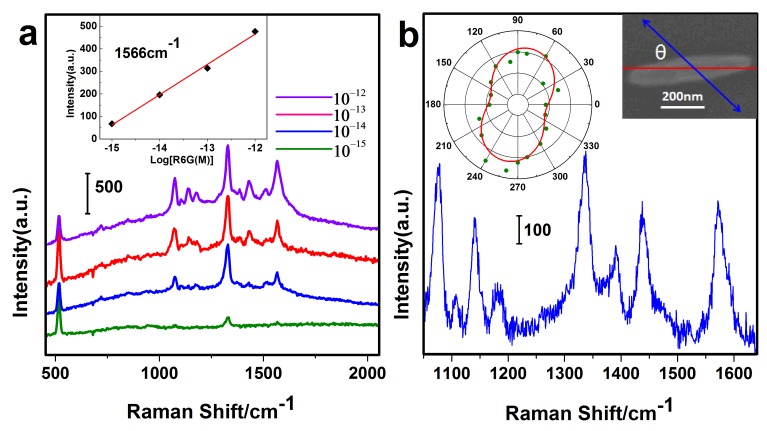
(**a**) Surface enhanced Raman scattering (SERS) spectra of R6G molecules with various concentrations. Inset: SERS intensity at 1566 cm^−1^ as a function of R6G concentrations; (**b**) SERS spectra of *p*,*p*′-dimercaptoazobenzene (DMAB) is collected at a single particle. Inset: Polar plot of the Raman intensity (solid green circles) versus the excitation polarization direction, the red curve is a fit to the cosine squared function (left) and the corresponding SEM image (right, θ represents the angle between polarization and horizontal).

Here, using the hollow Au–Ag alloy nanorices as an enhanced substrate, the SERS spectra of Rhodamine6G (R6G) molecules is shown in Figure 3a. The main Raman peaks appearing at 520 cm^−1^, 1074 cm^−1^, 1330 cm^−1^, and 1566 cm^−1^ are the characteristic Raman peaks of R6G molecule. With the decline in R6G molecular concentration, the Raman signals become gradually weak, while the main peaks still exist in virtue of the enhancement from the substrate. By analysis of SERS spectra of R6G with different concentrations, we also find that the SERS intensities of R6G at 1566 cm^−1^ follow a linear relation over the range of 10^−15^–10^−12^ (inset of Figure 3a). This linear relation can be expressed as I=134.5logC+2078.9, where C is the R6G concentration, and I is the SERS intensity. The detection limit is as low as 10^−15^ M under the experimental conditions.

We also further studied the PDSC of the hollow alloy nanorices. As is known, the surface plasmon causes the 4-Aminothiophenol (PATP) molecule to convert into a *p*,*p*′-dimercaptoazobenzene (DMAB) molecule, which is demonstrated by the appearance of three characteristic peaks 1143 cm^−1^, 1390 cm^−1^, and 1432 cm^−1^ of DMAB in the Raman spectra (Figure 3b). The PDSC reaction also has polarization dependence as the polar plot shown in Figure 3b. The Raman intensity is maximized when the polarization direction of the incident light is perpendicular to the nanorice, which is consistent with the previous report [35].

### 2.3. Oxidation Resistance of Nanorices

**Figure 4:**
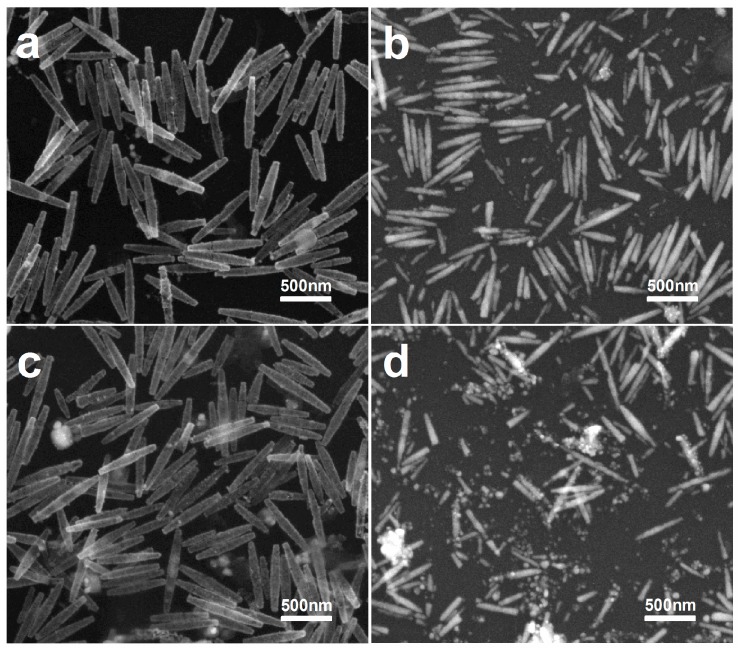
SEM of the alloy and Ag nanorices after being mixed with 3% aqueous H_2_O_2_ for different times. (**a**) Ten minutes (alloy nanorices); (**b**) Five minutes (Ag nanorices); (**c**) Five hours (alloy nanorices); (**d**) Ten minutes (Ag nanorices).

Furthermore, an oxidation test is performed whereby the hollow Au–Ag alloy nanorices are immersed into 3% aqueous H_2_O_2_ for 5 h. As shown in Figure 4c, the morphology of hollow Au–Ag alloy nanorices hardly change. By comparison, pristine Ag nanorices were mixed with the same aqueous H_2_O_2_ for 10 min (Figure 4d), but the morphology changed significantly. Therefore, the superior anti-oxidation property provides Au–Ag alloy nanorices with much broader application prospects.

## 3. Materials and Methods

### 3.1. Materials

Silver nitrate (99.99%), PVP (Mw = 58,000, K29-32), tetrachloroauric acid (99.9%), hydrogen peroxide (3 wt % solution in water), gold chloride trihydrate (99.99%), para-aminothiophenol (PATP), and R6G were purchased from Shanghai Aladdin biochemical Polytron Technologies Inc. (Shanghai, China). Polyethylene glycol 600 (PEG 600) was purchased from Chengdu Kelong Chemical Reagent Factory (Chengdu, China). All chemicals were used as received without further purification or treatment. High-purity deionized water (18.25 MΩ·cm) was produced using Aquapro AWL-0502-H (Aquapro International Company LLC., Dover, DE, USA).

### 3.2. Synthesis of Ag Nanorices

The Ag nanorices were synthesized based on modifying a previous synthetic method [21]. In this process, 0.2 mL of an AgNO_3_ aqueous solution (1 M) and 1 mL of a PVP aqueous solution (1 M) were respectively added to 10 mL of a PEG 600 solution in a 100 mL flask under stirring. Next, the flask was moved to an oil bath at a temperature of 110 °C via stirring for 10 h. After that, a gray silver colloid was obtained. The Ag nanorices can be gained from solution by centrifugation and washed with ethanol more than three times. Finally, the sample was dispersed in ethanol for further experimentation and characterization.

### 3.3. Synthesis of Hollow Au–Ag Alloy Nanorices

Hollow Au–Ag alloy nanorices were synthesized by a galvanic replacement reaction. In a typical synthesis, Ag nanorices were centrifuged from the silver colloid and subsequently redispersed in 4 mL of deionized water. Afterwards, the aqueous solution and 3 mL of PVP (0.5 M) were added to a flask. The flask was then transferred to an oil bath at a temperature of 90 °C under stirring, followed by the introduction of 2 mL of HAuCl_4_ solution (1 mM) using a syringe pump at a speed of 2 mL/h. Finally, hollow Au–Ag alloy nanorices were collected by centrifugation and washed with ethanol.

### 3.4. Characterization

The morphology of nanorices were characterized by TEM (Zeiss LIBRA 200 FEG) and SEM (TESCAN MIRA 3 FE), respectively. The optical properties were characterized by the dark-field scattering spectroscopy and surface enhanced Raman scattering spectroscopy.

### 3.5. Optical Properties Test

The dark-field experiment device was an inverted optical microscope fitted up with an oil immersion dark-field condenser (Olympus, 1.2–1.4 NA, (OLYMPUS (China) Co., Ltd., Shanghai, China)) shown in Figure S2a. White light generated by a halogen lamp was transmitted from the top to a transmission-type dark-field condenser. Next, scattering light of sample was collected using a 100× oil immersion objective (Olympus, 0.6–1.3 NA) and directed to a Charge-coupled Device. The nanorice particles diluted by ethanol were thrown on a conductive glass substrate evaporated in the air. Then, a commercial copper grid for marking position was attached to the sample to guarantee that dark-field spectra was from the nanorice labeled by SEM.

In a typical SERS analysis, hollow Au–Ag alloy nanorices with the same concentration and volume (5 μL) were deposited on a separate silicon substrate to form an enhanced basement. Then, 5 μL of aqueous solution of the R6G with different molar concentrations (10^−12^, 10^−13^, 10^−14^, and 10^−15^ M) were placed dropwise on the enhanced basement as a probe and dried in air, and the SERS activity was measured with the Raman spectrograph with a 633 nm He–Ne laser (3 mW). The signals were obtained with one scan every 20 s in all measurements.

In a typical PDSC analysis, adding hollow Au–Ag alloy nanorices to PATP molecules ethanol solutions (5 × 10^−6^ M) was stirred for more than 4 h to ensure that the molecules absorb on the surface of the nanorice. Then, these Ag nanorices were acquired by centrifuging to exclude the effect of the PATP molecules in the nanorice ethanol solution. The nanorices were then placed dropwise onto the clean silicon wafer, and Raman spectra were collected with a 633 nm laser.

### 3.6. Oxidation Resistance Analysis

Hollow Au–Ag alloy nanorices and Ag nanorices with the same volume were respectively immersed into 3% aqueous H_2_O_2_ for different times. The proper amount was then removed and added to the clean silicon to perform characterization.

### 3.7. COMSOL Multiphysics Simulation

The finite element method (COMSOL 5.2a commercial package) was used to calculate the dark-field scattering spectroscopy of an individual nanorice. In the simulation, all the nanorices were set as perfect ellipsoids surrounded by oil, while the propagating direction and polarization direction of incident light were aligned 60° and 30° with the long axis, respectively. The ellipsoids size was consistent with actual measurements throughout the calculation. In the simulation, the alloy shell was divided into several staggered Au and Ag layers, which is consistent with the previous report [36]. The permittivities of Au/Ag was adopted according to Johnson and Christy’s works [37].

## 4. Conclusions

In summary, a reliable synthetic protocol of hollow Au–Ag alloy nanorices is reported in this work. The high-resolution SEM and TEM images and corresponding EDS spectra demonstrated that the final product was hollow and alloy. Furthermore, the collected dark-field spectra and SERS of the hollow Au–Ag alloy nanorices, along with their good anti-oxidation properties, indicate that this novel metal nanostructure has broad application prospects in surface-plasmon-related fields, such as SERS, PDSC, multipolar plasmon-based antennas, and metamaterials.

## Supplementary Materials

The following are available online at http://www.mdpi.com/2079-4991/7/9/255/s1. Figure S1: experimented and simulated dark-field scattering spectra of nanorice. Figure S2: Schematic drawing of the dark-field setup and the geometry used in the simulations.
