# Supplementary material for: Hollow Au–Ag Alloy Nanorices and Their Optical Properties
Source: Nanomaterials (Basel). 2017 Sep 4;7(9):255. doi: 10.3390/nano7090255 (PMC5618366; doi:10.3390/nano7090255)
Supplement: Supplementary file 1 [file nanomaterials-07-00255-s001.pdf]

Supporting Information

Hollow Au-Ag Alloy Nanorices and Their Optical Properties

Keke Yu[1,2], Xiaonan Sun[1,*], Liang Pan[1], Ting Liu[1], Anping Liu[1], Guo Chen[2], Yingzhou Huang[2,*]

[1] Applied of Physics, College of Physics, Chongqing University, Chongqing, 400044, China, P. R. China

[2] Soft Matter and Interdisciplinary Research Center, College of Physics, Chongqing University, Chongqing, 400044, P. R. China

1. Experimented and Simulated Dark-Field Scattering Spectra of Nanorices

For the longer hollow nanorices (630 nm length), the peaks red-shift from 583 nm and 716 nm (hollow nanorice: 570 nm length) to 620 nm and 847 nm, respectively (Figure S1c). The plasmonic properties of hollow nanorices can also tuned by tuning the shell thickness. Figure S1d shows that, as the thickness of the shell decreases, the plasmon resonances red-shift and the resonant intensity increases, which is consistent with previous reports [1].

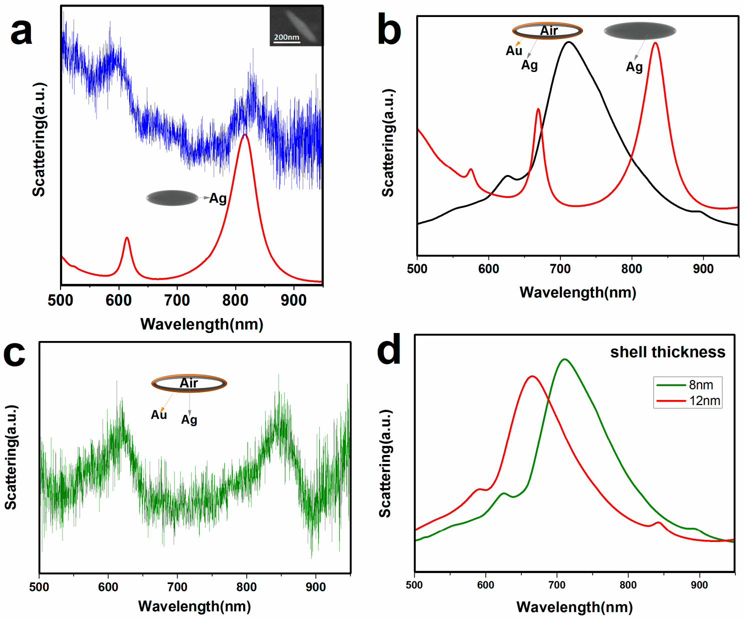

Figure S1. (**a**) Dark-field scattering spectra and corresponding simulation scattering spectra of Ag nanorices (with a 330 nm length). (**b**) The simulation scattering spectra with two different structures under the same simulated conditions. (**c**) Dark-field scattering spectra of the hollow Au–Ag alloy nanorices. (**d**) The simulation scattering spectra of hollow nanorices with different shell thicknesses.

2. Schematic Drawing of the Dark-Field Setup and the Geometry Used in Simulations

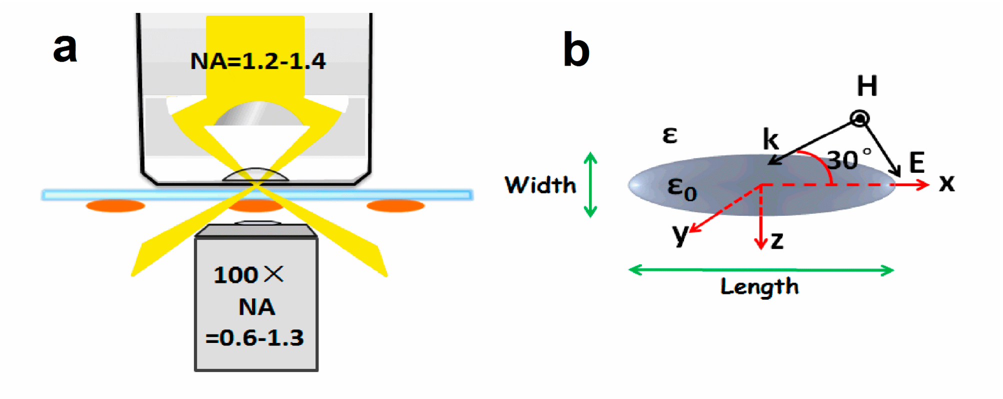

Figure S2. (**a**) Schematic of the dark-field setup; (**b**) schematic of the geometry used in simulations.

References

1. Zhang, Z.S.; Yang, Z.J.; Liu, X.L.; Li, M.; Zhou, L. Multiple plasmon resonances of Au/Ag alloyed hollow nanoshells. *Scripta Mater*. **2010**, *63*, 1193-1196.
